# Pectin Supplementation Improves Probiotic Survival and Preserves Bioactive Compounds of Fermented Pear Juice

**DOI:** 10.3390/foods15122200

**Published:** 2026-06-18

**Authors:** Dongsheng Niu, Daiyi Zhao, Aerzuguli Yalikun, Feng Li

**Affiliations:** College of Food Science and Pharmacy, Xinjiang Agricultural University, Urumqi 830052, China; 15124789941@163.com (D.N.); 18997873679@163.com (D.Z.); 18040765182@163.com (A.Y.)

**Keywords:** pectin, phenolic compounds, antioxidant capacity, probiotics, fermented pear juice

## Abstract

Fruit and vegetable juices are ideal probiotic carriers and pectin supplementation is promising for probiotic survival. In this study, we investigated the effects of high- and low-methoxyl pectin on *Lacticaseibacillus casei* 37 and *Lactobacillus helveticus* 76 in fermented pear juice (PJ) regarding fermentation, viability, and functionality. Our results showed that pectin protected probiotic viability at 4 °C for 28 days, with viable cell counts reaching 8.39–8.63 log colony-forming units/mL. Furthermore, it promoted phenolic compound release (e.g., gallic acid and protocatechuic acid), raising total phenolic content by 8.3–21.9% and total flavonoid content by 79.6–140.3%. It significantly enhanced DPPH, ABTS radical scavenging activity, and FRAP antioxidant capacity. In vitro digestion revealed that pectin supplementation elevated the survival rate of probiotics in simulated gastric juice by 6.2–66.4%. Additionally, correlation analysis linked specific phenolics (p-coumaric acid, epicatechin, rutin) to antioxidant activity. An addition of 0.3% low-methoxyl and 0.2% high-methoxyl pectin was considered the optimal treatment, benefiting probiotic viability, phenolic accumulation and antioxidant stability of fermented PJ under cold storage and gastrointestinal environment. Thus, pectin is an effective carrier for high-viability, high-antioxidant probiotic fermented PJ beverages.

## 1. Introduction

The market for functional foods and beverages rich in probiotics is rapidly expanding due to growing consumer demand for healthy diets. Probiotics exert beneficial effects on host health by regulating the gut microbiota balance, enhancing immunity, and promoting nutrient absorption [1]. As highlighted by Vicas [2], the stability and bioavailability of probiotics during digestion are critical for their functional efficacy, while appropriate food matrices and delivery systems are essential to ensure their survival and for health-promoting effects. However, a sufficient number of viable bacteria must withstand processing, storage, and the human gastrointestinal environment to colonize the intestines for probiotic products to deliver these health benefits [3]. Furthermore, probiotics must endure the digestive action of pancreatic enzymes and bile salts. This inevitably results in a significant reduction in viable counts, which severely compromises their probiotic efficacy [4]. As reported by Stachelska et al. [5], the sensitivity of probiotic lactic acid bacteria to gastrointestinal stressors (such as bile salts and digestive enzymes) is a key factor limiting their survival and functional effects in the host. Therefore, enhancing probiotic survival rates during processing, storage, and digestion is crucial for the development of effective probiotic products.

Fruit and vegetable juices are nutritionally rich, have pleasant flavors, and serve as ideal carriers for probiotics [6]. Particularly, pear juice (PJ) is rich in vitamins, minerals, and various bioactive compounds such as phenolic compounds, which have antioxidant properties [7]. However, compared to apple juice, orange juice, and grape juice—which are commonly utilized as fermentation substrates for probiotics—the initial pH of fresh PJ naturally ranges between 3.5 and 4.6, which is lower than that of most fruit juices [8]. Simultaneously, its primary components, such as easily fermentable glucose, fructose, and the unique sorbitol, continue to promote the accumulation of organic acids through fermentation during refrigeration. Furthermore, PJ is composed of high levels of arbutin, chlorogenic acid, and epicatechin—unique phenolic compounds that are extremely rare in other mainstream fruit juices; consequently, PJ presents particular challenges for probiotic survival compared to other fruit juices [7]. Lactic acid fermentation increases the value of PJ by releasing more bound phenolic compounds through biotransformation, thereby enhancing its antioxidant activity and generating distinctive flavors [9]. However, as reported by Wang et al. [10], compared with traditional dairy matrices, non-dairy matrices such as fruit juices exhibit more distinct nutritional compositions and physicochemical properties, posing unique challenges in maintaining probiotic viability and stability during processing, storage and gastrointestinal transit.

Immobilization of probiotics via physical adsorption onto pectin is an effective strategy for addressing these challenges. This method involves using fresh fruit and vegetable juices as the liquid matrix and pectin as a natural solid-phase adsorbent [11]. By leveraging electrostatic, hydrogen bonding, and hydrophobic interactions, it adsorbs and immobilizes lactic acid bacteria (probiotics) onto the surface of pectin macromolecules, thereby achieving in situ immobilization of the probiotics [12]. Low-methoxyl (LM) and high-methoxyl (HM) pectin vary remarkably in gelation behavior. LM pectin forms crosslinked gel networks primarily with divalent cations such as Ca^2+^ [13], whereas HM pectin gelatinizes under high-sugar and acidic environments [14]. Such fundamental discrepancy in gelling mechanisms largely accounts for their divergent protective effects on probiotics. Lu et al. [15] systematically classified cell immobilization technologies, and clarified that physical adsorption immobilization rely on electrostatic, hydrogen bonding and hydrophobic interactions to attach microbial cells onto carrier surfaces, establishing a theoretical basis for the technical route adopted in this study. As a typical physical adsorption cell immobilization technology, it differs from ionotropic crosslinking calcium pectin bead microencapsulation; nevertheless, both share pectin as the carrier material and rely on physical interactions to improve the stress tolerance and survival rate of probiotics [16]. In the study by Chackoshian Khorasani et al. [17], pectin was used as a natural biocompatible carrier matrix in a simulated gastrointestinal environment to protect probiotics in low-pH fruit juice. It was confirmed that pectin relies on electrostatic interaction and hydrogen bonding to construct a stable network structure that maintains probiotic viability [18]. This mechanism directly underlies the technical principle and application scenario of the pectin-adsorption immobilization strategy evaluated in this study.

Most existing relevant studies focus on single-category pectin cross-linking microcapsule encapsulation for probiotic protection in conventional apple, orange and grape juices, and have scarcely adopted in situ physical adsorption immobilization technology [19]. In addition, previous investigations mostly depended solely on one kind of pectin, limiting parallel comparison between HM and LM pectin within the same juice system [20]. Moreover, almost no studies explored pectin-mediated probiotic immobilization in characteristic PJ featured by unique sorbitol and special endogenous phenolics.

Addressing the above research limitations, the present study advances existing research by utilizing pectin physical adsorption immobilization to co-immobilize mixed *Lacticaseibacillus casei* 37 and *Lactobacillus helveticus* 76, comparatively exploring the functions of HM and LM pectin in PJ fermentation system. We also aimed to track successive variations in probiotic viability, organic acids, phenolic metabolites, and antioxidant capacity throughout the refrigerated storage and simulated gastrointestinal digestion in order to bridge the research gap regarding pectin-based probiotic immobilization in fermented PJ. The research objectives included: (1) assess how pectin physical adsorption immobilization affects probiotic viability, system acidity and organic acid metabolism of fermented PJ (FPJ) throughout refrigerated storage; (2) characterize the impacts of pectin physical adsorption immobilization on the compositional profile of phenolic compounds as the core research focus, alongside corresponding variations in in vitro antioxidant capacity; (3) explore the protective efficacy of pectin physical adsorption immobilization on probiotic cells under simulated gastrointestinal digestion, as well as resultant shifts in phenolic bioavailability and antioxidant activity. Collectively, this study provides theoretical basis and technical support for developing high-viability, stable novel probiotic-fortified fermented PJ beverages.

## 2. Materials and Methods

### 2.1. Activated Strains and Culture Preparations

Strain activation and culture preparation were performed as described previously [21]. *Lacticaseibacillus casei* 37 (Lc37) and *Lactobacillus helveticus 76* (Lh76) were provided by Shanghai Helplifes Technology Co., Ltd. (Shanghai, China) in lyophilized form. The bacterial strain was resuspended in MRS broth and incubated at 37 °C for 12 h. The pre-inoculum was then inoculated into MRS broth and cultivated at 37 °C for 12 h. The cultures were centrifuged at 5000× *g* for 5 min and washed twice with sterile normal saline to obtain the inoculum. All inoculation and strain transfer operations were completed on a SW CJ 1FD clean bench (Suzhou Antai Air Technology Co., Ltd., Suzhou, China). Sterilization of MRS medium and saline solution was implemented using a BXM 50VE vertical autoclave (Shandong Biobase Biological Industry Co., Ltd., Jinan, China).

### 2.2. Fermented Pear Juice (FPJ)

PJ was fermented as described previously [22]. Huangguan pears (*Pyrus bretschneideri* Rehd.) were rinsed with tap water, pulped with a PB12M1 701R juice extractor (Midea Group Co., Ltd., Foshan, China), and filtered through a 100-mesh sieve to yield raw PJ. LM and HM pectins were added to the juice, respectively, at concentrations of 0%, 0.1%, 0.2%, and 0.3% (*w*/*v*). The LM has a degree of esterification of 35%, while HM has 70%. Both of these pectins were purchased from Guangdong Oumi Chemical Reagent Co., Ltd. (Guangdong, China) The resulting samples were pasteurized at 80 °C for 5 min. For physical adsorption immobilization, 0.5% (*v*/*v*) mixed Lc37/Lh76 inoculum (1:1 volume ratio) was added to pasteurized PJ, shaken at 150 rpm for 20 min in an SPH 2102C shaking incubator (Shanghai Tiancheng Laboratory Instrument Manufacturing Co., Ltd., Shanghai, China) to fully mix and adhere to the surface of pectin. Subsequently, the samples were incubated at 37 °C for 48 h of fermentation. The fermented juice was stored at 4 °C for 28 days. In the current experimental design, the soluble solids content, pH, and titratable acidity of fresh Huangguang PJ were not quantified. Furthermore, no anti-browning treatments were applied during the peeling, dicing, and juicing of the pears. Throughout the experiments, no exogenous calcium ions were added to the PJ system to promote LM pectin gel formation. According to the protocol, the end of fermentation was defined as 48 h of static incubation at 37 °C. Fermentation was conducted in sealed, sterile conical flasks to prevent microbial contamination and loss of volatile compounds. Notably, we did not quantify the effects of pasteurization (at 80 °C for 5 min) on the phenolic compounds and endogenous pectin structural characteristics of the PJ.

### 2.3. Cryo Scanning Electron Microscopy

Cryo-scanning electron microscopy (SEM) (Nano SEM-450, FEI, Hillsboro, OR, USA) was employed to visualize the microstructure of fermented PJ with and without pectin according to Tie et al. [23]. The samples were dropped on copper sample holders and rapidly frozen in liquid nitrogen. Thereafter, the resulting samples were sublimated and coated with platinum in the preparation chamber. Subsequently, samples were imaged using SEM at −140 °C. All cryo-scanning electron microscopy images were acquired at a magnification of 24,000× and an acceleration voltage of 5.00 kV, which is consistent with the parameters indicated on the original SEM images. For each sample, at least six independent fields of view were randomly observed and imaged. The micrographs ultimately presented in the figure were objectively selected as representative images that reflected the general microstructural characteristics of the corresponding samples, rather than isolated abnormal regions.

### 2.4. Simulated Gastrointestinal Digestion

The in vitro digestion was performed according to Valero-Cases et al. [24] with some modifications. Briefly, 30 mL juice was added to 120 mL PBS (pH 3 adjusted with 1 M HCl) with 3 g/L pepsin, then incubated at 37 °C at 130 rpm for 60 min for simulated gastric digestion (SGD). The pH of the resulting sample was adjusted to 7.0 with 1 M NaHCO_3_, followed by addition of bile salts (4.5 g/L) and pancreatin (1 g/L), and finally incubated at 37 °C at 50 rpm for 120 min for simulated intestinal digestion (SID).

### 2.5. Determination of Viable Counts in Fermented Pear Juice

The viable counts were determined using a plate counting method according to Valero-Cases et al. [24]. Serial dilutions of FPJ sampled across storage time points and post-digestion stages were spread onto MRS agar plates, incubated at 37 °C for 48 h in an LRH 250CL low-temperature incubator (Shanghai Yiheng Scientific Instrument Co., Ltd., Shanghai, China). All live bacterial counts were converted and expressed in colony-forming units (CFU) per milliliter of the original, undiluted fermented PJ.

### 2.6. Determination of Titratable Acidity and Organic Acids

The titratable acidity was determined following the AOAC official method (AOAC 942.15) via titration with 0.01 M NaOH and expressed as the percentage of lactic acid. Organic acid profiles were quantified by high-performance liquid chromatography (HPLC, LC-20A, Shimadzu, Kyoto, Japan) following Zhou et al. [25]. Organic acids were eluted on a reversed-phase C18 column (4.6 mm × 250 mm, 5 μm, Waters, Milford, MA, USA) with 0.01 M KH_2_PO_4_-H_3_PO_4_ (pH 2.7)/methyl alcohol (97/3, *v*/*v*) at 0.6 mL/min. The injection volume was 10 μL and detection wavelength was 210 nm. Absorbance readings for titration endpoint judgment were recorded using a UV 1780 ultraviolet–visible spectrophotometer (Shimadzu, Japan).

### 2.7. Determination of Phenolic Compounds

The total phenolic content (TPC) and total flavonoid content (TFC) were measured with Folin–Ciocalteu and AlCl_3_ colorimetry, respectively [26,27]. All colorimetric absorbance values were detected on the UV 1780 UV–vis spectrophotometer (Shimadzu, Japan). The TPC was determined using the Folin–Ciocalteu method. Herein, 0.5 mL of diluted phenolic extracts was added to 2.5 mL Folin–Ciocalteu (10%, *v*/*v*) for 3 min of incubation, and 2 mL of 7.5% (*w*/*v*) Na_2_CO_3_ solution was added. Then, the resulting solution was incubated in the dark for 60 min and the absorbance at 765 nm was detected. TPC was calculated as gallic acid equivalents (mg GAE/L). The TFC was determined using AlCl_3_ colorimetry. Briefly, 0.5 mL of 50 g/L NaNO_2_ solution was added to 4 mL of diluted PJ for 5 min of incubation. Thereafter, 1 mL of 100 g/L AlCl_3_ solution was added for 5 min of incubation, and 2 mL of 2 mol/L NaOH was added for 10 min of incubation. The absorbance at 510 nm was measured. The TFC was calculated by the rutin equivalent (mg RE/L). Individual phenolic monomers were qualitatively and quantitatively profiled by HPLC (LC-20A, Shimadzu, Japan) with modified conditions from Ren et al. [28] with some modifications. Phenolic compounds were separated on a reversed-phase C18 column (4.6 mm × 250 mm, 5 μm, Waters, USA) with injection volume of 10 μL. The mobile phase was composed of solvent A (0.1% formic acid) and solvent B (acetonitrile). The gradient elution was conducted as follows: 0% B at 0 min, 5% B at 5 min, 12% B at 25 min, 30% B at 40 min, 45% B at 50 min, and 5% B at 60 min. The flow-rate was 1 mL/min, and detection wavelength was 280 nm.

### 2.8. Determination of Antioxidant Capacity (AOC)

#### 2.8.1. DPPH Free Radical Scavenging Ability

DPPH (2,2-diphenyl-1-picrylhydrazyl) free radical scavenging ability was determined according to Z. Wang et al. [29]. Accordingly, 2 mL of diluted PJ was added to 4 mL of DPPH solution and incubated for 30 min in dark. The absorbance was detected at 517 nm, and the results were expressed as the percentage of DPPH radical inhibition.

#### 2.8.2. ABTS Radical Scavenging Ability

ABTS (2,2′-azino-bis(3-ethylbenzothiazoline-6 sulfonic acid)) radical scavenging ability was determined according to Zeng et al. [30]. Briefly, K_2_S_2_O_8_ (2.45 mM) was mixed with ABTS (7 mM) at 1:1 (*v*/*v*), and kept in the dark for 16 h, followed by dilution with 80% ethanol to an absorbance of 0.70 at 734 nm to obtain the ABTS working solution. The diluted juice (0.6 mL) was added to the ABTS solution (5.4 mL), and incubated for 6 min, and then absorbance at 734 nm was detected. The results were expressed as the percentage of ABTS radical inhibition.

#### 2.8.3. Ferric Reducing Antioxidant Power (FRAP)

FRAP was determined according to Valero-Cases et al. [24]. Herein, 0.3 M acetate buffer (pH 3.6), 10 mM TPTZ, and 20 mM FeCl_3_ were mixed at 10:1:1 (*v*/*v*/*v*) and incubated at 37 °C for 30 min to obtain the FRAP solution. Subsequently, the diluted juice (0.2 mL) was mixed with FRAP solution (6 mL), and incubated at 37 °C for 30 min, following which absorbance at 593 nm was detected. The results were expressed as Trolox equivalent.

### 2.9. Statistical Analysis

All of the experiments were performed at least in triplicate. Statistical analysis was conducted on SPSS software (Version 26.0, IBM Corporation, Armonk, NY, USA). One-way analysis of variance (ANOVA) and Tukey’s test was performed to determine the significant differences (*p* < 0.05).

## 3. Results and Discussion

### 3.1. Probiotic Viability and Fermentation Parameters of Pear Juice

#### 3.1.1. Probiotic Viability During Storage

**Figure 1:**
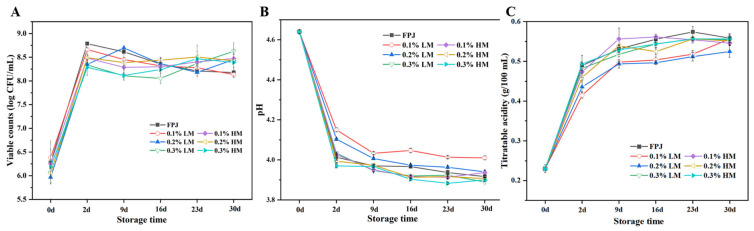
Viable counts (**A**), pH (**B**), and titratable acidity (**C**) in fermented juice with and without pectin during storage.

Figure 1A shows the live cell count of the probiotics after 48 h of fermentation followed by cold-chain storage. Following the fermentation, the viable counts were significantly increased to 8.28–8.78 log CFU/mL, thereby suggesting that PJ (with and without pectin) was a favorable medium for probiotics (Lc37/Lh76) and that it can serve as a probiotic functional beverage (*p* < 0.05). The viable count in FPJ without pectin decreased from 8.78 log CFU/mL on day 0 to 8.18 log CFU/mL during storage. However, for FPJ with pectin (except for 0.1% LM), the viable counts remained stable or slightly increased during storage, and the viable counts after 28 days (8.63–8.39 log CFU/mL) were significantly higher than those of FPJ without pectin (8.18 log CFU/mL). These results preliminarily suggest that the resulting pectin network structure benefits the maintenance of continuous metabolic activity of probiotics (Lc37/Lh76) during low-temperature storage. This is supported by higher viable cell counts and elevated levels of organic acids, such as citric acid and lactic acid, in pectin-supplemented groups. Collectively, these metabolic changes imply that pectin matrix could support a microenvironment conducive to probiotic survival during refrigeration-based storage.

#### 3.1.2. pH, Titratable Acidity, and Organic Acids During Storage

pH dramatically decreased and titratable acid significantly increased after fermentation for all samples (Figure 1B,C). FPJ with and without pectin showed similar trends in pH and titratable acidity during storage. After 30 days, pH decreased to 3.89–4.01 and titratable acid increased to 0.52–0.56 g/100 mL for FPJ with pectin, thereby showing no significant difference compared to FPJ without pectin (pH 3.92 and titratable acid of 0.56 g/100 mL). These results indicate that post-acidification occurred during low-temperature storage owing to continued fermentation [31]. Notably, sensory evaluation was not performed in the present work; thus, whether the final pH and titratable acidity levels satisfy acceptable sensory quality of fermented pear beverage needs further verification in the follow-up research.

Changes in pH and titratable acidity are directly related to organic acids, which are metabolites of lactic acid bacteria (LAB) that play crucial roles in the flavor and storage of fermented juice [32]. Table S1 shows the organic acid profiles of unfermented and fermented juice during storage at 4 °C. Malic and tartaric acid contents in PJ were markedly reduced after fermentation, whereas citric and lactic acid levels were significantly increased. Moreover, the oxalic acid content of FPJ with pectin was significantly higher than that of FPJ without pectin. During storage, lactic acid in FPJ showed no significant differences, whereas it showed slight (0.1% LM and 0.3% HM) or significant (0.2% LM, 0.3% LM, 0.1% HM, and 0.2% HM) increases in FPJ with pectin. Although citric acid in FPJ with and without pectin increased significantly, the increase in FPJ with pectin (1.5 to 10.80 times) was dramatically greater than that in FPJ without pectin (1.19 times) after 28 days (*p* < 0.05). The elevated organic acids after metabolism of fructose, glucose, and sucrose (the main carbohydrates in PJ) by LAB reflected the vitality of the probiotic culture [33]. These results preliminarily imply that the supplemented pectin matrix sustained probiotic metabolic activity during the refrigeration; nevertheless, organic acid accumulation is jointly regulated by the available carbon substrate, pH condition, and storage temperature, apart from bacterial vitality.

#### 3.1.3. Cryo-SEM Analysis of Fermented Pear Juice

**Figure 2:**
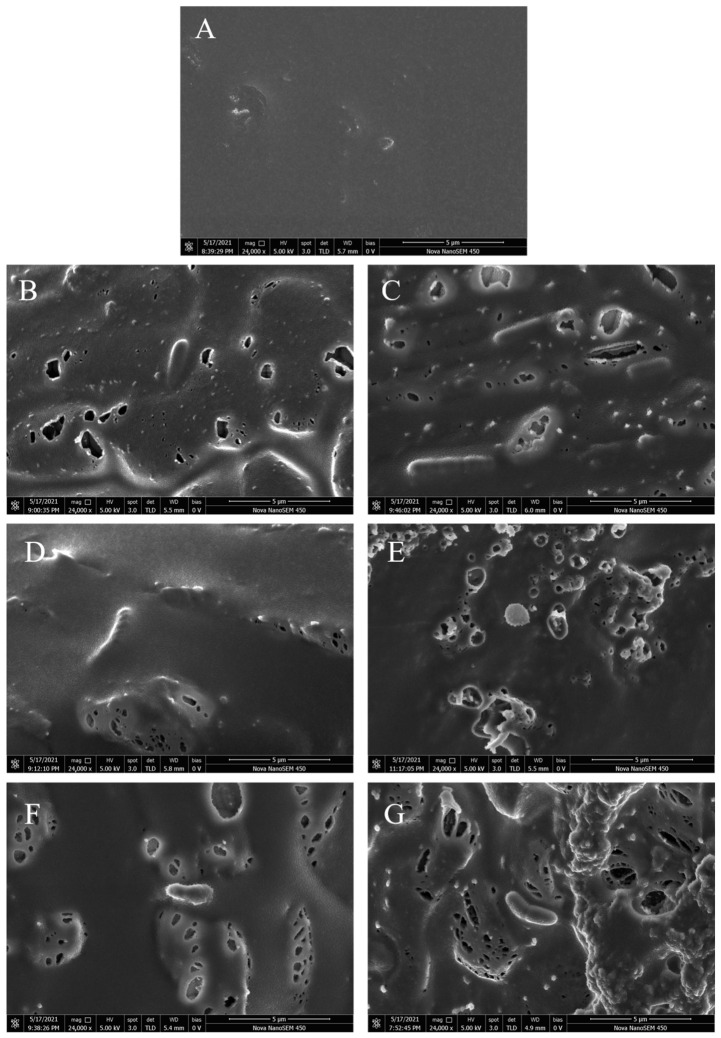
Cryo-SEM images of fermented pear juice (PJ) with and without pectin: (**A**) fermented juice without pectin; (**B**,**D**,**F**) fermented PJ with 0.1% LM (**B**), 0.2% LM (**D**), and 0.3% LM (**F**); (**C**,**E**,**G**) fermented PJ with 0.1% HM (**C**), 0.2% HM (**E**), and 0.3% HM (**G**). Note: LM and HM represent low-methoxyl pectin and high-methoxyl pectin, respectively.

Micrograph images of the probiotics and pectin in FPJ are shown in Figure 2. No gel network structure was observed in FPJ owing to the low pectin content of PJ. However, all FPJ with pectin exhibited a network structure with numerous protrusions and pores, and numerous probiotics attached to this structure. This may be attributed to the excellent cell entrapment of pectin owing to its small pore size [34]. Thus, the gel network formed by the encapsulated bacterial cells is expected to enhance the probiotics’ tolerance to low temperatures. A similar result was previously reported; *L. plantarum* immobilization in natural and pectin-supplemented apple juice was beneficial to bacterial survival during storage at 4 °C [35].

### 3.2. Effect of Pectin Immobilization on the Phenolic Compounds in FPJ

**Figure 3:**
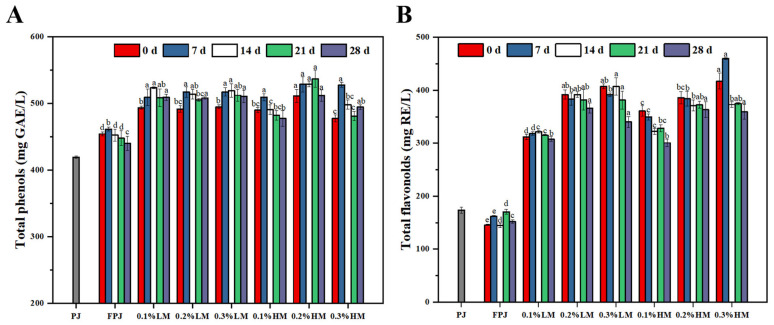
Total phenols (**A**) and flavonoids (**B**) in fermented pear juice with and without pectin during storage. The gray bars represent unfermented raw pear juice (PJ, used solely as an initial baseline control); different lowercase letters at the tops of the bars within the same treatment group indicate significant differences between groups (*p* < 0.05).

Phenolic compounds, which are important bioactive compounds in pears, can scavenge free radicals and prevent various chronic diseases [36]. The release of phenolic compounds from the food matrix improves their bioaccessibility [37]. Figure 3A,B show the effects of pectin immobilization on the TPC and TFC of FPJ during storage. TPC was significantly increased (8.3–21.9%) after fermentation, and the TPC of fermented juice with pectin was significantly higher (5.1–12.5%) than that of FPJ without pectin. During storage, TPC first increased and then decreased, and the TPC in FPJ with pectin was significantly higher than that in FPJ without pectin (*p* < 0.05). Compared with the control PJ, TFC decreased significantly (by 15.9%) in FPJ without the added pectin, whereas this increase peaked significantly (by 79.6–140.3%) in FPJ with the added pectin (*p* < 0.05). Similar to the TPC, the TFC in FPJ with pectin was remarkably higher than that in FPJ without pectin during storage. LAB fermentation promotes the release of conjugated phenolic compounds from the cell wall of the substrate, which may result in an increase in TPC and TFC in FPJ [38]. These results suggest that pectin immobilization enhances the release of phenolic compounds from FPJ, which may be attributed to the protection of probiotics by pectin.

Phenolic compounds in PJ primarily include phenolic acids, flavonoids, and phenolic glycosides [39]. Ten phenolic compounds were identified and quantified, namely, five phenolic acids, two flavonoids, and three phenolic glycosides (Table S2). Arbutin and epicatechin were the predominant phenolic compounds in unfermented juice, with arbutin and protocatechuic acid becoming the predominant phenolic compounds after fermentation; arbutin was also the most abundant phenolic compound during storage. The sharp decrease in epicatechin after fermentation was likely due to microbial enzyme-induced degradation, where epicatechin was metabolized into protocatechuic acid by fermentation-derived oxidases [40]. Arbutin increased in FPJ without (1.09%) and with 0.1% (18.77%) and 0.3% (4.32%) LM compared to unfermented juice. Arbutin levels significantly decreased during storage in most fermented samples; however, they significantly increased in fermented juice with 0.3% LM and 0.1% HM, with the content in fermented juice with 0.3% LM being the highest (539.90 mg/L) after 28 days. Arbutin is an important tyrosinase inhibitor. Bacteria produce arbutin by glycosylation biosynthesis, and low pH induces arbutin degradation [41]. Changes in arbutin in PJ before and after fermentation and during storage may result from the biotransformation by probiotics in combination with degradation at low pH. The relatively higher arbutin content in FPJ with 0.3% LM after 28 days could be related to the highest viable counts (8.63 log CFU/mL). As a potent tyrosinase inhibitor, the elevated arbutin content in pectin-supplemented groups partly accounts for the improved antioxidant capacity observed in DPPH, ABTS and FRAP assays.

Chlorogenic acid was the predominant phenolic acid, which increased after fermentation (4.20–9.48%; except for 0.1% LM) and showed no significant differences among fermented juices. The chlorogenic acid levels remained stable in FPJ with and without LM pectin during storage, whereas it was not detected in juice with HM after 28 days. These results suggest that the chlorogenic acid in PJ may be a product and substrate of probiotic metabolism. Additionally, gallic and protocatechuic acid content significantly increased to 8.38–21.40% and 106.50–241.57% (except for 0.1% pectin) after fermentation, respectively. The content of both phenolic acids increased during storage, and after 28 days, the content in FPJ with pectin was higher than that in FPJ without pectin (3.40–17.74% and 54.81–147.10% for gallic and protocatechuic acid, respectively). The accumulation of these phenolic acids in pectin groups is consistent with the higher antioxidant activity observed in corresponding fermented juice.

In terms of flavonoids, the rutin levels were not significantly different before and after fermentation or during storage. Epicatechin increased only in FPJ with 0.3% HM (14.97%) and without HM (15.91%) in comparison with unfermented juice. After 28 days, only the content in fermented juice with 0.3% pectin was higher (5.44–9.53%) than that in FPJ without pectin. Various enzymes produced by LAB participate in the bioconversion of phenolic compounds. β-glucosidase promotes the release of bound phenolics. Meanwhile, tannase hydrolyzes epigallocatechin gallate into gallic acid and catalyzes the conversion of catechins and gallic acid to protocatechuic acid [42]. The results of this study are consistent with those of a previous study [43] which reported that *Lactobacillus* fermentation increased gallic and protocatechuic acid levels in blueberries. These results confirm that pectin immobilization significantly influences the metabolism of LAB, which changes the phenolic compounds in FPJ. The fluctuating epicatechin also contributes to variation in antioxidant capacity during refrigerated storage.

### 3.3. Effect of Pectin Immobilization on the Antioxidant Capacity of Fermented Pear Juice

**Figure 4:**
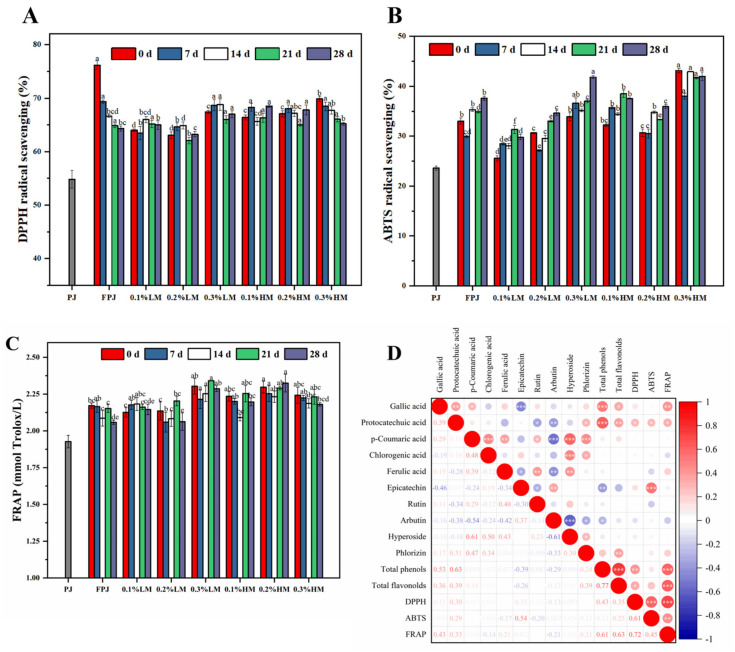
Antioxidant capacity (AOC) of fermented juice with and without pectin during storage and correlation of AOC with phenolic compounds. DPPH (**A**); ABTS (**B**); FRAP (**C**); Heat map of Pearson correlation coefficient of phenolics profile and AOC (**D**). * Correlation is significant at *p* < 0.05; ** Correlation is significant at *p* < 0.01; *** Correlation is significant at *p* < 0.001. Circle size: The diameter of each circle is proportional to the absolute value of the correlation coefficient |*r*|. Larger circles correspond to stronger correlation (larger |*r*|), while smaller circles represent weaker correlation (smaller |*r*|). The diagonal solid red circles show self-correlation of each indicator (*r* = 1, maximum circle size). The gray bars represent unfermented raw pear juice (PJ, used solely as an initial baseline control); different lowercase letters at the tops of the bars within the same treatment group indicate significant differences between groups (*p* < 0.05).

The changes in the AOC of PJ fermented with and without pectin during storage are shown in Figure 4. DPPH and ABTS scavenging activities and FRAP significantly increased after fermentation with and without pectin. FPJ without pectin had a higher DPPH scavenging activity than FPJ with pectin. However, the DPPH scavenging activity of FPJ continuously decreased during storage and was dramatically lower than that of FPJ with pectin (0.3% LM, 0.1% HM, and 0.2% HM) after 28 days of storage (Figure 4A). The ABTS scavenging activity of FPJ with and without pectin increased during storage, and the ABTS scavenging activity of FPJ with pectin (0.3% LM and 0.3% HM) was significantly higher than that of FPJ after 28 days of storage (Figure 4B). FRAP values of FPJ with and without pectin fluctuated during storage, and after 28 days of storage, the FRAP of FPJ with pectin (except for 0.2% LM) was significantly higher than that of FPJ without pectin (Figure 4C). These results indicate that LAB fermentation enhances the AOC of PJ, and that pectin immobilization at an appropriate concentration may enhance the AOC of FPJ during storage. The differences in AOC between the three evaluation methods may be attributed to the different antioxidant mechanisms involved in hydrogen atom transfer and electron donation [44].

Phenolic compounds are important antioxidants, and the biotransformation of phenolics influences the AOC [45]. Therefore, a Pearson’s correlation analysis was performed to assess the potential contribution of individual phenolic compounds to each AOC evaluation method [46]. A significant positive correlation was observed between ABTS scavenging activity and epicatechin content (r = 0.55, *p* < 0.01) (Figure 4D). FRAP was significantly positively correlated with the gallic (r = 0.40, *p* < 0.01) and protocatechuic acid contents (r = 0.33, *p* < 0.05). These results suggest that the transformation of phenolic compounds induced by LAB fermentation could be responsible for changes in the AOC of FPJ during storage.

### 3.4. Probiotic Viability During in Vitro Digestion

**Figure 5:**
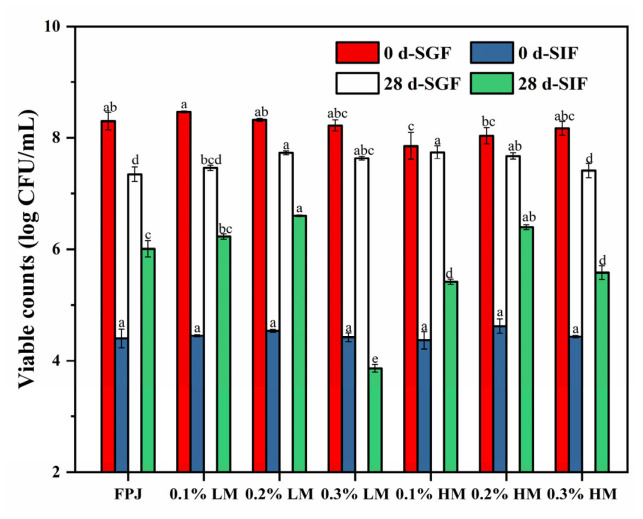
Viable counts in fermented pear juice with and without pectin (at 0 and 28 d) during in vitro digestion. SGF: Simulated Gastric Fluid; SIF: Simulated Intestinal Fluid; LM: Low-methoxyl pectin; HM: High-methoxyl pectin; FPJ: Fermented Pear Juice. Different lowercase letters at the tops of the bars within the same treatment group indicate significant differences between groups (*p* < 0.05).

An in vitro simulated gastrointestinal digestion (SGD) system is commonly employed to evaluate the survival potential of probiotics and their suitability for fermented carriers [47]. Figure 5 shows the effects of in vitro digestion on the viability of FPJ before and after storage. After SGD, the viable counts in fermented juice without pectin significantly decreased from 8.78 log CFU/mL to 8.30 log CFU/mL (*p* < 0.05), whereas no significant reductions were observed in fermented juice with pectin (except for juices with 0.1% and 0.2% HM) (*p* > 0.05). These results align with those of Roberts et al. [35], who found that the viable counts with pectin immobilization in apple juice were significantly higher than those of juice with free cells during SGD. This finding may be attributed to the fact that pectin physical adsorption immobilization protects bacteria against the low pH of SGD [48]. Conversely, its protective effect against intestinal bile is relatively limited and only effective for specific pectin treatments after 28-day storage. The viable counts in fermented juice were dramatically decreased after 28 days of storage, with the counts in FPJ with pectin (0.2% LM, 0.3% LM, 0.1% HM, and 0.2% HM) being significantly higher than those in FPJ without pectin (6.2–66.4%). The higher viable counts in juice containing pectin may be because more cells were immobilized on pectin over time, and the decreased pH caused by post-acidification improved the stability of pectin [49].

After simulated intestinal digestion (SID), the viable counts were remarkably reduced (16.5–49.6%). The viable counts were also reduced after 28 days of storage (14.7–24.7%), except for juice with 0.3% LM, which showed a smaller reduction in viable counts than that before storage (42.5–47.4%). Bile salt stress may be a key factor influencing the viability loss of probiotics, and refrigerated storage could improve their tolerance to bile salts [50]. No significant differences were observed in viable counts for FPJ with and without pectin at 0 days. However, after 28 days of storage, the counts in FPJ with pectin (0.1% LM, 0.2% LM, and 0.2% HM) were significantly higher than those of FPJ without pectin. Pectin exerted marked protection against gastric acid, while its protective role against bile salt was restricted and observed in partial pectin-supplemented samples after storage.

### 3.5. Effect of Pectin Immobilization on the Phenolic Compounds of Fermented Pear Juice During In Vitro Digestion

**Figure 6:**
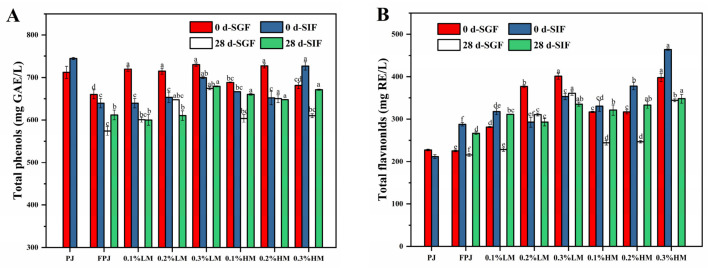
Total phenols (**A**) and flavonoids (**B**) in fermented pear juice with and without pectin (at 0 and 28 d) during in vitro digestion. SGF: Simulated Gastric Fluid; SIF: Simulated Intestinal Fluid; LM: Low-methoxyl pectin; HM: High-methoxyl pectin; FPJ: Fermented Pear Juice. Different lowercase letters at the tops of the bars within the same treatment group indicate significant differences between groups (*p* < 0.05).

The TPC and TFC in FPJ at 0 and 28 days of storage after in vitro digestion are shown in Figure 6. Compared to undigested juices, the TPC was significantly increased during SGD (17.9–69.8%) and SID (17.8–77.5%) (Figure 6A). The TPC in fermented juice at 28 days was lower than that at 0 days after SGD (9.4–16.5%) and SID (3.0–9.3%). The TPC in FPJ with pectin was significantly higher than that of FPJ without pectin after SGD (3.2–10.6% at 0 days and 4.6–17.5% at 28 days) and SID (2.0–13.7% at 0 days and 1.1–9.7% at 28 days), except for 0.1% and 0.2% LM, which showed no significant difference. During SGD, the low pH and pepsin content could contribute to the hydrolysis of the ester bonds between phenolic acids and proteins, which resulted in the release of free phenols [51]. The decrease in TPC after 28 days of storage may be because LAB metabolism changes the composition of phenolic compounds during storage, thereby making them easier to degrade and transform during in vitro digestion.

Similarly, the TFC in unfermented juice and FPJ without pectin significantly increased during SGD and SID, whereas the TFC in FPJ with pectin decreased (Figure 6B). However, the TFC in FPJ with pectin was significantly higher than that of FPJ without pectin during SGD (25.0–78.4% at 0 days and 5.7–67.4% at 28 days) and SID (1.8–61.4% at 0 days and 9.6–30.4% at 28 days). Free flavonoids are easily degraded and oxidized because they are sensitive to acidic or basic environments during in vitro digestion [52]. Thus, although SGD and SID enhanced the release of free flavonoids, the reduction in TFC in FPJ with pectin was likely caused by the degradation/oxidation of free flavonoids being greater than their release during SGD and SID.

Furthermore, 10 major phenolic compounds in FPJ (at 0 and 28 days) were measured to investigate their stability during in vitro digestion (Table S3). In unfermented PJ, arbutin was significantly reduced (17.6%) after SGD and remarkably increased (30.7%) after SID. Arbutin can be hydrolyzed into hydroquinone at a low pH during SGD [53]. However, the arbutin content in unfermented juice after SID (532.34 mg/L) was higher than that of undigested juice (494.71 mg/L), thereby suggesting that the arbutin bond was released during SID. In FPJ without pectin, arbutin was reduced after SGD (53.9% at 0 days and 23.1% at 28 days), significantly increased after SID at 0 days (14.8%), and decreased after 28 days of storage (47.5%). In FPJ with pectin, arbutin was increased (0.5–189.6% at 0 days and 12.0% at 28 days) or decreased (12.9–36.7% at 0 days and 0.6–51.2% at 28 days) after SGD, and dramatically decreased (58.3–100% at 0 days and 85.9–100% at 28 days) after SID. The arbutin content in FPJ with pectin was significantly lower than that in juice without pectin after digestion. These results suggest that bacterial arbutin metabolism occurred during in vitro digestion, and pectin may influence the transformation of arbutin by LAB.

The bacterial metabolism of phenolics during in vitro digestion of pomegranate juice was previously reported [24]. Protocatechuic acid, a major phenolic compound in fermented juices, was not detected after digestion because of its degradation. Gallic acid in unfermented juice showed no significant difference after SGD but dramatically increased (38.0%) after SID. In FPJ with and without pectin, gallic acid levels decreased after SGD (2.1–69.3% at 0 days and 20.7–58.6% at 28 days), and showed an increase or no significant difference in most samples after SID. The fermented juice with 0.1% LM exhibited the highest gallic acid content after digestion (52.35 mg/L at 0 days and 41.54 mg/L at 28 days). Gallic acid in pomegranate juice fermented with *L. acidophilus* was dramatically reduced after SGD compared to other LABs [24]. Thus, the decrease in gallic acid content could be related to LAB metabolism during SGD. Additionally, the increase in gallic acid content may be attributed to gallotannin hydrolysis during SIG. In unfermented juice, SGD contributed to 5.0-, 3.5-, and 4.9-fold increases in the release of chlorogenic acid, epicatechin, and rutin, respectively. Meanwhile, chlorogenic acid and epicatechin were not detected, and rutin showed no significant difference after SID. Similarly, a remarkable increase in chlorogenic acid, epicatechin, and rutin was observed in the partially fermented samples during SGD/SID, whereas these compounds were not detected in the other samples. The remarkable increase or disappearance of these phenolic compounds could be due to the following reasons: digestive enzymes promoted the release of phenolics in PJ; bacterial metabolism transformed phenolics; and instability at high pH and interactions between phenolics and digestive mixtures [24].

### 3.6. Effect of Pectin Immobilization on the Antioxidant Capacity of Fermented Pear Juice During In Vitro Digestion

**Figure 7:**
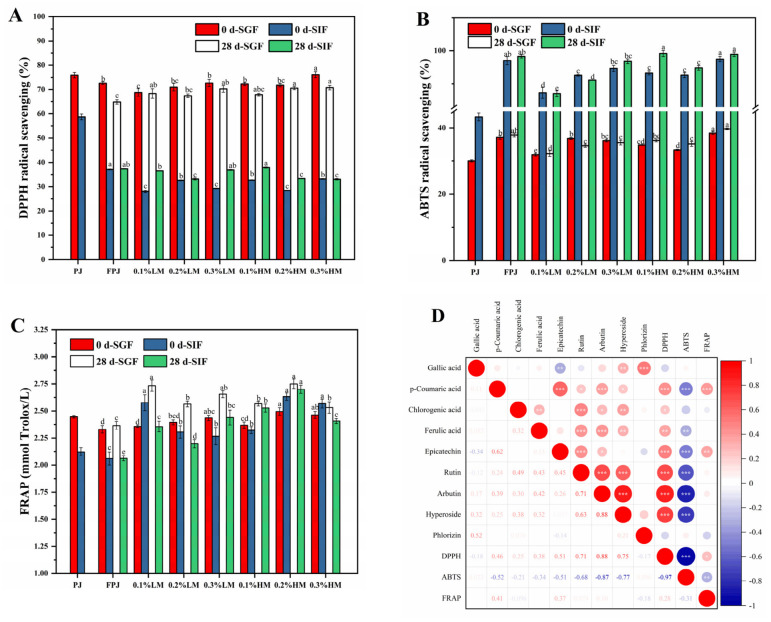
AOC of fermented juice with and without pectin (at 0 and 28 d) during in vitro digestion and correlation of AOC with phenolic compounds. DPPH (**A**) and ABTS (**B**) scavenging activities and FRAP (**C**). Heat map of Pearson correlation coefficient of phenolics profile and AOC (**D**). * Correlation is significant at *p* < 0.05; ** Correlation is significant at *p* < 0.01; *** Correlation is significant at *p* < 0.001. SGF: Simulated Gastric Fluid; SIF: Simulated Intestinal Fluid; LM: Low-methoxyl pectin; HM: High-methoxyl pectin; FPJ: Fermented Pear Juice. Circle size: The diameter of each circle is proportional to the absolute value of the correlation coefficient |*r*|. Larger circles correspond to stronger correlation (larger |*r*|), while smaller circles represent weaker correlation (smaller |*r*|). The diagonal solid red circles show self-correlation of each indicator (*r* = 1, maximum circle size). Different lowercase letters at the tops of the bars within the same treatment group indicate significant differences between groups (*p* < 0.05).

Phenolic compounds can be transformed through isomerization, degradation, oxidation, and hydrolysis during fermentation and digestion, thereby altering their structure and AOC [54]. DPPH scavenging activities of unfermented and fermented juices were increased after SGD (7.0–35.5%, except for FPJ at 0 days) and decreased after SID (22.7–60.4%) (Figure 7A). After digestion, the DPPH scavenging activity of FPJ (27.99–37.92%) was significantly lower than that of unfermented juice (54.83%). Fermented juice with 0.3% HM showed the highest DPPH scavenging activity after SGD, whereas its activity was significantly lower than that of FPJ after SID. This phenomenon was primarily attributed to the optimal protective effect of 0.3% HM pectin on bound phenolic substances under gastric acidic condition; acid hydrolysis promoted massive release of free phenolic antioxidants in this group, contributing to elevated DPPH radical scavenging capacity. Nevertheless, the released active phenolics were susceptible to degradation under the neutral/weak alkaline intestinal environment, leading to evident activity decline after subsequent intestinal digestion. The reduction in DPPH scavenging activity after SID may be due to the lower reactivity of the corresponding antioxidant compounds, which resulted from their low stability in simulated intestinal environments [55]. ABTS scavenging activity remained unchanged or slightly decreased after SGD, whereas it significantly increased after SID (44.4–177.6%) (Figure 7B). The fermented juice with 0.3% HM had the highest ABTS scavenging activity after SGD (38.4% at 0 days and 39.7% at 28 days) and SID (97.4% at 0 days and 98.9% at 28 days). Trypsin enhances the electron-donating ability, which may improve the ABTS scavenging activity [56]. For unfermented juice and FPJ, the FRAP increased after SGD and decreased after SID (Figure 7C). Similar tendencies were observed in most FPJ with pectin, except for juices with 0.1% LM, 0.2% HM, and 0.3% HM at 0 days (showing an increase after SID). The FRAP of FPJ with pectin was higher than that of FPJ without pectin after SGD (1.2–7.1% at 0 days and 7.0–16.3% at 28 days) and SID (10.0–27.7% at 0 days and 6.5–30.9% at 28 days). FPJ with 0.2% HM showed the highest FRAP during SGD (2.49 and 2.75 mmol Trolox/L at 0 and 28 days, respectively) and SID (2.63 and 2.70 mmol Trolox/L at 0 and 28 days, respectively). These results indicate that pectin immobilization improves the FRAP AOC of FPJ during in vitro digestion. The increase in FRAP after SGD can be attributed to the electron transfer reaction under acidic conditions [55]. Compared with FPJ without pectin, the higher AOC in the FRAP of FPJ with pectin may be related to the increase in the chelating activity of bacterial metabolites [57,58].

DPPH was significantly positively correlated with the contents of p-coumaric acid (r = 0.64, *p* < 0.001), epicatechin (r = 0.51, *p* < 0.001), rutin (r = 0.71, *p* < 0.001), arbutin (r = 0.88, *p* < 0.001), hyperoside (r = 0.75, *p* < 0.001), ferulic acid (r = 0.38, *p* < 0.01), and chlorogenic acid (r = 0.25, *p* < 0.05) (Figure 7D). In contrast, ABTS was significantly negatively correlated with individual phenols (except for gallic acid, chlorogenic acid, and phloridzin). FRAP was significantly positively correlated with p-coumaric acid (r = 0.41, *p* < 0.001) and epicatechin (r = 0.37, *p* < 0.01). During simulated gastrointestinal digestion, most parent phenolic compounds degrade and convert into derivatives with weaker ABTS radical-scavenging ability; some intermediate degradation by-products competitively consume ABTS radicals, resulting in reduced ABTS activity alongside decreasing phenolic concentrations. These results demonstrate that the transformation of phenolic compounds during in vitro digestion plays a crucial role in the AOC of PJ. In addition, obvious positive inter-correlation was found among DPPH, ABTS and FRAP values, as shown in Figure 7D. DPPH exhibited a strong positive correlation with ABTS and FRAP, revealing consistently changing trends of radical-scavenging capacity, as determined by the three methods during simulated digestion. Such positive linkage indicates that the variation in phenolic composition synchronously regulates multiple antioxidant pathways including hydrogen-donating and ferric-reducing reactions. Nevertheless, inconsistent correlation trends between individual phenolics and different antioxidant indices (positive for DPPH/FRAP while mostly negative for ABTS) implied distinct antioxidant reaction mechanisms of phenolic constituents toward diverse free radical systems. Taken together, these results demonstrate that the transformation of phenolic compounds during in vitro digestion crucially influences the AOC of PJ.

## 4. Conclusions

This study explored the application of supplemental pectin in fermented probiotic PJ. Pectin supplementation, particularly 0.3% LM and 0.2% HM, showed favorable potential to improve probiotic survival and metabolic performance under refrigerated storage conditions. Furthermore, the added pectin facilitated the transformation and accumulation of organic acids and phenolic constituents, which enhances the overall AOC of FPJ. Pectin addition also mitigated damage to probiotic cells during simulated gastric digestion and partially preserved their intestinal tolerance after storage. Additionally, the dynamic variations in phenolics during in vitro digestion were closely correlated with changes in antioxidant activity. In conclusion, supplementation with selected pectin types and dosages possesses robust potential to optimize probiotic viability and the functional attributes of fermented PJ; nevertheless, further investigations, including immobilization efficiency quantification, sensory assessment, in vivo bioavailability testing, and commercial shelf-life evaluation, are necessary to support its practical industrial development as a functional beverage.

## Data Availability

The original contributions presented in this study are included in the article/Supplementary Material. Further inquiries can be directed to the corresponding author.

## Supplementary Materials

The following supporting information can be downloaded at https://www.mdpi.com/article/10.3390/foods15122200/s1, Table S1 Organic acids in fermented juice with and without pectin during storage process. Table S2 Phenolics profile in fermented pear juice with and without pectin during storage process. Table S3 Phenolics profile in fermented pear juice with and without pectin during (at 0 d and 28 d) in vitro digestion.

## Abbreviations

The following abbreviations are used in this manuscript:

ABTS	2,2′-Azino-bis(3-ethylbenzothiazoline-6-sulfonic acid)
AOC	Antioxidant capacity
cryo-SEM	Cryo scanning electron microscopy
DPPH	2,2-Diphenyl-1-picrylhydrazyl
FPJ	Fermented pear juice
FRAP	Ferric reducing antioxidant power
LAB	Lactic acid bacteria
HM	High-methoxyl
LM	Low-methoxyl
PJ	Pear juice
SGD	Simulated gastric digestion
SID	Simulated intestinal digestion
HPLC	High-performance liquid chromatography
TPC	Total phenolic content
TFC	Total flavonoid content
