# Supplementary material for: Pectin Supplementation Improves Probiotic Survival and Preserves Bioactive Compounds of Fermented Pear Juice
Source: Foods. 2026 Jun 18;15(12):2200. doi: 10.3390/foods15122200 (PMC13298052; doi:10.3390/foods15122200)
Supplement: Supplementary file 1 [file foods-15-02200-s001.zip › foods-4352488-supplementary.pdf]

Pectin Supplementation Improves Probiotic Survival and Preserves Bioactive Compounds of Fermented Pear Juice

Dongsheng Niu, Daiyi Zhao, Aerzuguli•yalikun, Feng Li*

College of Food Science and Pharmacy, Xinjiang Agricultural University, China

author:

Dongsheng Niu(First author)

E-mail: 15124789941@163.com

Daiyi Zhao

E-mail: 18997873679@163.com

Aerzuguli•yalikun

E-mail: 18040765182@163.com

Feng Li (Corresponding author)

Tel/Fax: +86 18089209017

E-mail: 18089209017@163.com

Table S1 Organic acids in fermented juice with and without pectin during storage process.

Organic acids (mg/mL)	Storage time (d)	PJ	FPJ	0.1%LM	0.2%LM	0.3%LM	0.1%HM	0.2%HM	0.3%HM
Oxalic acid	0	0.33 ± 0.01^{Ab}	0.28 ± 0.01^{Ac}	0.33 ± 0.01^{Ab}	0.33 ± 0.01^{Ab}	0.32 ± 0.00^{Ab}	0.32 ± 0.01^{Ab}	0.36 ± 0.01^{Aa}	0.29 ± 0.00^{Ac}
	14		0.28 ± 0.02^{Ad}	0.30 ± 0.01^{Bbc}	0.30 ± 0.00^{Bab}	0.30 ± 0.01^{Abc}	0.28 ± 0.00^{Bc}	0.32 ± 0.01^{Ba}	0.29 ± 0.01^{Ac}
	28		0.29 ± 0.01^{Abc}	0.29 ± 0.01^{Bbc}	0.30 ± 0.01^{Bab}	0.30 ± 0.02^{Aa}	0.28 ± 0.01^{Bc}	0.30 ± 0.01^{Bb}	0.27 ± 0.01^{Bd}
Tartaric acid	0	0.18 ± 0.01^{Ac}	ND	0.35 ± 0.01^{Ab}	0.32 ± 0.01^{Ab}	0.39 ± 0.01^{Ab}	0.38 ± 0.01^{Ab}	1.94 ± 0.07^{Aa}	ND
	14		ND	ND	0.34 ± 0.04^{Aab}	ND	ND	0.32 ± 0.01^{Bb}	0.39 ± 0.01^{Aa}
	28		ND	0.30 ± 0.02^{Ba}	ND	ND	ND	ND	ND
Malic acid	0	1.25 ± 0.02^{Aa}	ND	ND	ND	ND	ND	ND	ND
	14		ND	ND	ND	ND	ND	ND	ND
	28		ND	ND	ND	ND	ND	ND	ND
Lactic acid	0	0.01 ± 0.00^{Ad}	10.98 ± 0.64^{Aa}	8.93 ± 0.10^{Ac}	8.82 ± 0.60^{Bc}	9.61 ± 0.47^{Cc}	9.75 ± 0.19^{Bbc}	9.64 ± 0.41^{Bbc}	10.82 ± 0.47^{Aab}
	14		11.37 ± 0.32^{Aab}	9.25 ± 0.22^{Ac}	9.74 ± 0.31^{ABc}	10.67 ± 0.12^{Bb}	10.74 ± 0.20^{Ab}	10.67 ± 0.55^{Ab}	11.61 ± 0.39^{Aa}
	28		10.73 ± 0.21^{Aab}	9.29 ± 0.16^{Ac}	10.35 ± 0.14^{Ab}	11.17 ± 0.19^{Aa}	10.94 ± 0.29^{Aab}	10.71 ± 0.48^{Aab}	11.07 ± 0.47^{Aab}
Citric acid	0	0.13 ±0.00^{Ae}	0.70 ± 0.02^{Ba}	0.68 ± 0.02^{Ba}	0.48 ± 0.02^{Bb}	0.40 ± 0.03^{Bcd}	0.36 ± 0.02^{Cd}	0.44 ± 0.03^{Cbc}	0.05 ± 0.00^{Bf}
	14		0.69 ± 0.02^{Bb}	1.07 ± 0.08^{Aa}	0.50 ± 0.02^{Bd}	0.64 ± 0.03^{Abc}	0.54 ± 0.03^{Bd}	0.68 ± 0.02^{Bbc}	0.58 ± 0.02^{Acd}
	28		0.83 ± 0.02^{Abc}	1.19 ± 0.16^{Aa}	0.72 ± 0.04^{Acd}	0.64 ± 0.04^{Ade}	0.91 ± 0.01^{Ab}	0.81 ± 0.04^{Abcd}	0.54 ± 0.03^{Ae}

Significant differences ($p < 0.05$) in the same row are indicated by superscripted lowercase letters, and significant differences ($p < 0.05$) during different storage periods are indicated by capital letters. ND means “not detected”.

Table S2 Phenolics profile in fermented pear juice with and without pectin during storage process.

Phenolic compound （mg/L）	Storage time (d)	CK	FPJ	0.1% LM	0.2% LM	0.3% LM	0.1% HM	0.2% HM	0.3% HM
Gallic acid	0	36.77 ± 2.79^{b}	39.85 ± 1.37^{Aab}	43.35 ± 2.86^{Aa}	44.64 ± 1.74^{Ba}	43.25 ± 1.10^{ABa}	44.40 ± 1.32^{Aa}	43.47 ± 0.68^{Aa}	40.05 ± 2.00^{ABab}
	14		39.00 ± 0.69^{Aabc}	42.92 ± 1.94^{Aa}	41.92 ± 1.06^{Bab}	39.69 ± 2.19^{Babc}	35.53 ± 2.19^{Bc}	42.75 ± 1.69^{Aa}	39.06 ± 1.13^{Babc}
	28		40.81 ± 1.03^{Abc}	46.63 ± 0.84^{Aab}	48.05 ± 0.99^{Aa}	47.81 ± 2.66^{Aa}	42.20 ± 1.25^{Abc}	45.68 ± 2.46^{Aabc}	43.16 ± 1.56^{Aabc}
Protocatechuic acid	0	20.47 ± 1.40^{d}	42.27 ± 0.72^{Bc}	ND	69.92 ± 2.33^{Ca}	74.78 ± 1.80^{Ca}	23.76 ± 1.78^{Cd}	69.03 ± 1.43^{Ca}	57.99 ± 2.68^{Cb}
	14		42.92 ± 2.13^{Bd}	121.76 ± 2.28^{Aa}	93.60 ± 3.11^{Bb}	91.17 ± 0.72^{Bb}	73.31 ± 1.25^{Bc}	93.27 ± 1.78^{Bb}	74.04 ± 1.64^{Bc}
	28		51.34 ± 0.36^{Ae}	126.86 ± 2.54^{Aa}	101.97 ± 2.07^{Ac}	100.55 ± 1.56^{Ac}	103.48 ± 2.59^{Ac}	109.18 ± 2.33^{Ab}	79.48 ± 1.88^{Ad}
P-coumaric acid	0	1.36 ± 0.20^{b}	1.50 ± 0.31^{Ab}	ND	1.18 ± 0.21^{Ab}	1.76 ± 0.29^{Ab}	4.35 ± 0.32^{Aa}	1.90 ± 0.54^{Ab}	1.49 ± 0.40^{Bb}
	14		1.36 ± 0.17^{Ab}	1.96 ± 0.21^{Aab}	1.95 ± 0.65^{Aab}	1.30 ± 0.24^{ABb}	1.31 ± 0.27^{Bb}	1.27 ± 0.12^{Ab}	2.55 ± 0.19^{Aa}
	28		1.71 ± 0.11^{Aab}	1.90 ± 0.19^{Aa}	1.93 ± 0.06^{Aa}	1.18 ± 0.03^{Bc}	ND	1.91 ± 0.11^{Aa}	1.37 ± 0.13^{Bbc}
Chlorogenic acid	0	40.92 ± 2.13^{a}	42.64 ± 0.41^{Aa}	ND	43.57 ± 0.43^{Aa}	43.18 ± 0.68^{Aa}	43.42 ± 1.74^{Aa}	44.80 ± 2.84^{Aa}	43.69 ± 1.65^{Aa}
	14		43.39 ± 1.52^{Aa}	44.92 ± 1.96^{Aa}	45.12 ± 1.09^{Aa}	43.94 ± 0.93^{Aa}	42.90 ± 2.01^{Aa}	42.96 ± 2.27^{Aa}	42.16 ± 0.83^{Aa}
	28		42.90 ± 0.16^{Aab}	41.70 ± 0.55^{Aab}	44.69 ± 0.89^{Aa}	40.82 ± 1.03^{Bb}	ND	ND	ND
Ferulic acid	0	1.49 ± 0.11^{b}	1.45 ± 0.17^{Ab}	1.48 ± 0.19^{Ab}	1.27 ± 0.12^{Ab}	1.14 ± 0.15^{Ab}	3.05 ± 1.07^{Aa}	1.65 ± 0.31^{Bb}	1.23 ± 0.09^{Ab}
	14		1.23 ± 0.08^{Aa}	1.06 ± 0.19^{ABb}	1.23 ± 0.06^{Aa}	1.07 ± 0.15^{Ab}	1.21 ± 0.06^{Ba}	1.26 ± 0.14^{Ba}	1.01 ± 0.10^{Bb}
	28		1.39 ± 0.16^{Ab}	1.19 ± 0.08^{Bbc}	1.30 ± 0.07^{Ab}	1.18 ± 0.04^{Abc}	1.43 ± 0.11^{Bb}	2.35 ± 0.20^{Aa}	0.94 ± 0.04^{Bc}

Continued

Phenolic compound （mg/L）	Storage time (d)	CK	FPJ	0.1% LM	0.2% LM	0.3% LM	0.1% HM	0.2% HM	0.3% HM
Epicatechin	0	54.43 ± 0.66^{b}	63.09 ± 0.94^{Aa}	ND	ND	42.31 ± 1.32^{Bc}	ND	30.51 ± 1.15^{Ad}	62.58 ± 2.90^{Aa}
	14		58.72 ± 1.79^{Ba}	30.39 ± 1.46^{Ab}	30.96 ± 1.53^{Ab}	59.00 ± 2.78^{Aa}	34.21 ± 0.72^{Ab}	30.41 ± 1.73^{Ab}	57.15 ± 1.42^{Ba}
	28		52.22 ± 1.35^{Cb}	ND	30.61 ± 1.13^{Ac}	57.20 ± 0.35^{Aa}	32.44 ± 2.03^{Ac}	31.96 ± 1.80^{Ac}	55.06 ± 1.83^{Bab}
Rutin	0	39.05 ± 1.08^{ab}	39.95 ± 0.84^{Aab}	40.26 ± 2.58^{Aab}	39.66 ± 0.25^{Aab}	39.91 ± 0.46^{Aab}	41.91 ± 1.02^{Aa}	38.28 ± 0.11^{Ab}	39.03 ± 0.66^{Aab}
	14		39.28 ± 0.30^{Aa}	39.17 ± 0.39^{Aa}	38.26 ± 0.09^{Ba}	38.26 ± 0.39^{Ba}	38.17 ± 0.01^{Ba}	38.47 ± 1.52^{Aa}	38.37 ± 0.55^{Aa}
	28		38.70 ± 0.48^{Aa}	38.65 ± 0.35^{Aa}	39.06 ± 0.58^{ABa}	38.44 ± 0.75^{Ba}	38.33 ± 1.45^{Ba}	39.78 ± 2.75^{Aa}	38.16 ± 0.34^{Aa}
Arbutin	0	494.71 ± 1.86^{cd}	500.12 ± 2.89^{Ac}	587.57 ± 7.09^{Aa}	490.38 ± 2.93^{Acd}	516.12 ± 2.37^{Bb}	174.92 ± 1.44^{Cf}	378.85 ± 3.70^{Ae}	485.54 ± 2.95^{Ad}
	14	494.71 ± 1.86^{a}	453.12 ± 2.63^{Bbc}	301.58 ± 3.04^{Bf}	450.01 ± 3.08^{Bc}	479.83 ± 1.07^{Ca}	375.71 ± 2.09^{Bd}	352.82 ± 2.33^{Be}	459.51 ± 4.42^{Bb}
	28		425.96 ± 2.87^{Cc}	293.50 ± 1.99^{Be}	450.01 ± 7.87^{Bb}	532.90 ± 7.01^{Aa}	439.38 ± 1.90^{Ab}	359.48 ± 3.92^{Bd}	445.14 ± 4.25^{Cb}
Hyperoside	0	5.86 ± 0.77^{c}	8.48 ± 0.17^{Ab}	2.30 ± 0.12^{Bd}	2.05 ± 0.16^{Bd}	0.89 ± 0.14^{Bd}	14.48 ± 0.73^{Aa}	6.79 ± 0.78^{Ac}	6.99 ± 0.46^{Ac}
	14		6.38 ± 0.30^{Babc}	5.63 ± 0.43^{Abc}	6.90 ± 0.53^{Aa}	6.02 ± 0.54^{Aabc}	5.19 ± 0.18^{Bc}	6.45 ± 0.23^{Aab}	5.86 ± 0.11^{Babc}
	28		6.88 ± 0.72^{Ba}	6.20 ± 0.11^{Aa}	7.01 ± 0.13^{Aa}	1.57 ± 0.27^{Bc}	1.97 ± 0.11^{Cc}	3.39 ± 0.30^{Bb}	4.19 ± 0.13^{Cb}
Phlorizin	0	0.16 ± 0.02^{b}	ND	ND	0.27 ± 0.07^{Bab}	0.27 ± 0.06^{Aab}	0.33 ± 0.07^{Aa}	0.28 ± 0.03^{Aab}	0.22 ± 0.06^{Aab}
	14		0.17 ± 0.02^{Ab}	0.19 ± 0.03^{Ab}	0.52 ± 0.03^{Aa}	0.19 ± 0.02^{Ab}	0.15 ± 0.03^{Bb}	0.21 ± 0.08^{Ab}	0.17 ± 0.02^{Ab}
	28		0.19 ± 0.03^{Aa}	0.21 ± 0.03^{Aa}	0.19 ± 0.02^{Ba}	0.18 ± 0.03^{Aa}	0.20 ± 0.01^{Ba}	0.20 ± 0.02^{Aa}	0.20 ± 0.02^{Aa}

Significant differences ($p < 0.05$) in the same row are indicated by superscripted lowercase letters, and significant differences ($p < 0.05$) during different storage periods are indicated by capital letters. ND means “not detected”.

Table S3 Phenolics profile in fermented pear juice with and without pectin during (at 0 d and 28 d) in vitro digestion.

Phenolic compound（mg/L）	Storage time（d）	Digestion	CK	FPJ	0.1%LM	0.2%LM	0.3%LM	0.1%HM	0.2%HM	0.3%HM
Gallic acid	0	SDG	37.87 ± 1.93^{Bb}	12.25 ± 1.37^{Dd}	42.46 ± 2.31^{Ba}	21.35 ± 0.59^{Dc}	38.81 ± 1.93^{Aab}	38.58 ± 1.68^{Aab}	39.11 ± 0.93^{Bab}	38.38 ± 0.79^{Aab}
		SID	52.26 ± 1.57^{Aa}	37.99 ± 0.81^{Ab}	52.35 ± 1.85^{Aa}	34.27 ± 0.79^{Abc}	33.82 ± 1.64^{Bc}	37.00 ± 1.82^{Abc}	49.44 ± 1.06^{Aa}	37.33 ± 1.55^{Abc}
	28	SGD	37.87 ± 1.93^{Ba}	32.38 ± 1.89^{Bab}	35.87 ± 1.78^{Ca}	31.11 ± 0.26^{Bb}	30.07 ± 1.83^{Bb}	30.72 ± 1.14^{Bb}	33.46 ± 2.37^{Cab}	17.87 ± 0.95^{Cc}
		SID	52.26 ± 1.57^{Aa}	28.14 ± 0.31^{Cd}	41.54 ± 1.46^{Bb}	28.83 ± 0.87^{Cd}	31.33 ± 0.48^{Bcd}	29.83 ± 1.81^{Bd}	33.48 ± 1.39^{Cc}	29.57 ± 1.15^{Bd}
P-coumaric acid	0	SGD	5.67 ± 0.36^{Aa}	ND	ND	ND	ND	ND	ND	ND
		SID	5.70 ± 0.44^{Aa}	ND	ND	ND	ND	ND	ND	ND
	28	SGD	5.67 ± 0.36^{Aa}	ND	5.72 ± 0.30^{Aa}	5.74 ± 0.22^{Aa}	5.66 ± 0.18^{Aa}	5.73 ± 0.05^{Aa}	5.87 ± 0.24^{Aa}	ND
		SID	5.70 ± 0.44^{Aa}	ND	ND	ND	ND	ND	ND	ND
Chlorogenic acid	0	SGD	204.30 ± 6.65^{Aa}	203.80 ± 3.61^{Aa}	204.57 ± 3.31^{Aa}	ND	204.07 ± 3.18^{Aa}	204.17 ± 1.90^{Aa}	204.47 ± 1.99^{Aa}	204.06 ± 3.40^{Aa}
		SID	ND	203.90 ± 2.82^{Aa}	ND	203.85 ± 5.72^{Aa}	203.92 ± 2.83^{Aa}	ND	ND	203.97 ± 3.48^{Aa}
	28	SGD	204.30 ± 6.65^{Aa}	204.08 ± 2.23^{Aa}	ND	203.76 ± 2.27^{Aa}	ND	203.68 ± 1.82^{Aa}	203.88 ± 2.28^{Aa}	ND
		SID	ND	ND	ND	203.83 ± 3.34^{Aa}	204.32 ± 1.51^{Aa}	ND	ND	204.02 ± 2.18^{Aa}
Ferulic acid	0	SGD	4.58 ± 0.40^{Aa}	4.71 ± 0.66^{Aa}	ND	ND	4.96 ± 0.27^{Aa}	4.48 ± 0.40^{Aa}	4.29 ± 0.17^{Aa}	4.47 ± 0.40^{Aa}
		SID	4.46 ± 0.43^{Aa}	4.35 ± 0.29^{Aa}	4.29 ± 0.23^{Aa}	4.39 ± 0.30^{Aa}	ND	ND	ND	4.40 ± 0.10^{Aa}
	28	SGD	4.58 ± 0.40^{Aa}	ND	ND	ND	ND	ND	4.54 ± 0.12^{Aa}	4.86 ± 0.17^{Aa}
		SID	4.46 ± 0.43^{Aa}	ND	ND	ND	ND	ND	ND	ND
Epicatechin	0	SGD	188.24 ± 2.33^{Aa}	ND	ND	154.98 ± 2.23^{Ab}	ND	ND	ND	ND
		SID	ND	ND	ND	ND	ND	ND	ND	ND

Continued

Phenolic compound（mg/L）	Storage time（d）	Digestion	CK	FPJ	0.1%LM	0.2%LM	0.3%LM	0.1%HM	0.2%HM	0.3%HM
Epicatechin	28	SGF	188.24 ± 2.33^{Aa}	ND	ND	154.12 ± 1.24^{Ac}	162.90 ± 0.95^{Ab}	156.11 ± 1.25^{Ac}	154.51 ± 2.42^{Ac}	160.19 ± 4.08^{Abc}
		SIF	ND	ND	ND	ND	ND	ND	ND	ND
Rutin	0	SGD	189.77 ± 1.92^{Aa}	188.88 ± 2.52^{Aa}	189.21 ± 1.69^{Aa}	189.76 ± 3.91^{Aa}	189.60 ± 4.03^{Aa}	189.52 ± 3.29^{Aa}	189.36 ± 4.01^{Aa}	189.15 ± 5.02^{Aa}
		SID	190.01 ± 8.47^{Aa}	189.47 ± 2.35^{Aa}	ND	ND	189.59 ± 0.90^{Aa}	ND	ND	189.52 ± 2.37^{Aa}
	28	SGD	189.77 ± 1.92^{Aa}	189.37 ± 2.88^{Aa}	ND	189.10 ± 3.05^{Aa}	189.49 ± 3.07^{Aa}	189.09 ± 1.53^{Aa}	189.95 ± 3.49^{Aa}	189.42 ± 3.09^{Aa}
		SID	190.01 ± 8.47^{Aa}	ND	ND	ND	191.71 ± 1.14^{Aa}	ND	ND	ND
Arbutin	0	SGD	407.40 ± 3.51^{Bc}	230.43 ± 12.89^{Ce}	511.84 ± 4.95^{Aab}	310.54 ± 3.46^{Bd}	518.73 ± 7.24^{Aa}	506.60 ± 4.85^{Aab}	513.05 ± 4.13^{Aab}	495.48 ± 2.93^{Ab}
		SID	532.34 ± 2.85^{Aa}	264.54 ± 2.39^{Bb}	24.80 ± 0.68^{Dd}	14.33 ± 0.57^{Ce}	ND	ND	21.15 ± 0.64^{Dd}	206.42 ± 1.56^{Cc}
	28	SGD	407.40 ± 3.51^{Ba}	327.76 ± 3.28^{Ad}	328.58 ± 3.27^{Bd}	334.33 ± 2.48^{Acd}	361.87 ± 3.72^{Bb}	342.75 ± 1.90^{Bc}	357.15 ± 5.70^{Bb}	217.44 ± 1.98^{Be}
		SID	532.34 ± 2.85^{Aa}	172.11 ± 1.62^{Db}	46.38 ± 0.54^{Cc}	ND	6.50 ± 0.02^{Cf}	ND	43.53 ± 0.63^{Cd}	14.40 ± 0.19^{De}
Hyperoside	0	SGD	3.97 ± 0.48^{Ab}	1.49 ± 0.08^{Bc}	4.93 ± 0.32^{Aa}	0.87 ± 0.02^{Bc}	3.58 ± 0.05^{Ab}	3.93 ± 0.18^{Ab}	3.76 ± 0.21^{Ab}	4.05 ± 0.07^{Ab}
		SID	4.27 ± 0.24^{Aa}	ND	ND	ND	0.70 ± 0.05^{Cc}	0.50 ± 0.02^{Cc}	0.45 ± 0.03^{Cc}	1.09 ± 0.10^{Bb}
	28	SGD	3.97 ± 0.48^{Aa}	4.20 ± 0.01^{Aa}	2.13 ± 0.04^{Bb}	1.54 ± 0.10^{Acd}	1.30 ± 0.29^{Bd}	1.73 ± 0.08^{Bc}	1.36 ± 0.12^{Bcd}	0.77 ± 0.05^{Ce}
		SID	4.27 ± 0.24^{Aa}	ND	ND	ND	0.38 ± 0.04^{Db}	ND	ND	ND
Phlorizin	0	SGD	0.66 ± 0.04^{Ba}	ND	0.76 ± 0.08^{Aa}	ND	ND	0.71 ± 0.03^{Aa}	ND	ND
		SID	0.84 ± 0.09^{Aab}	0.73 ± 0.02^{Ab}	0.78 ± 0.04^{Aab}	ND	0.80 ± 0.04^{Aab}	ND	0.87 ± 0.02^{Aa}	ND
	28	SGD	0.66 ± 0.04^{Ba}	ND	ND	ND	ND	ND	ND	ND
		SID	0.84 ± 0.09^{Aa}	ND	ND	ND	ND	0.18 ± 0.02^{Ab}	ND	0.72 ± 0.10^{Aa}

Significant differences ($p < 0.05$) in the same row are indicated by superscripted lowercase letters, and significant differences ($p < 0.05$) during different storage periods are indicated by capital letters. ND means “not detected”.
